# Transient Modulation of Working Memory Performance and Event-Related Potentials by Transcranial Static Magnetic Field Stimulation over the Dorsolateral Prefrontal Cortex

**DOI:** 10.3390/brainsci11060739

**Published:** 2021-06-02

**Authors:** Xiaoxiao Chen, Tatsunori Watanabe, Nami Kubo, Keisuke Yunoki, Takuya Matsumoto, Takayuki Kuwabara, Toru Sunagawa, Shota Date, Tatsuya Mima, Hikari Kirimoto

**Affiliations:** 1 Department of Sensorimotor Neuroscience, Graduate School of Biomedical and Health Sciences, Hiroshima University, Hiroshima 734-8553, Japan; d185984@hiroshima-u.ac.jp (X.C.); e.orinoco26@gmail.com (N.K.); d205546@hiroshima-u.ac.jp (K.Y.); d203652@hiroshima-u.ac.jp (T.M.); m203434@hiroshima-u.ac.jp (T.K.); hkirimoto@hiroshima-u.ac.jp (H.K.); 2 Japan Society for the Promotion of Science, Tokyo 102-0083, Japan; 3 Department of Analysis and Control of Upper Extremity Function, Graduate School of Biomedical and Health Sciences, Hiroshima University, Hiroshima 734-8553, Japan; torusuna@hiroshima-u.ac.jp (T.S.); sdate@hiroshima-u.ac.jp (S.D.); 4 Graduate School of Core Ethics and Frontier Sciences, Ritsumeikan University, Kyoto 603-8577, Japan; t-mima@fc.ritsumei.ac.jp

**Keywords:** transcranial static magnetic field stimulation, non-invasive brain stimulation, working memory, dorsolateral prefrontal cortex, event-related potentials, N2, P3

## Abstract

Transcranial static magnetic field stimulation (tSMS) can modulate human cortical excitability and behavior. To better understand the neuromodulatory effect of tSMS, this study investigates whether tSMS applied over the left dorsolateral prefrontal cortex (DLPFC) modulates working memory (WM) performance and its associated event-related potentials (ERPs). Thirteen healthy participants received tSMS or sham stimulation over the left DLPFC for 26 min on different days. The participants performed a 2-back version of the *n*-back task before, during (20 min after the start of stimulation), immediately after, and 15 min after the stimulation. We examine reaction time for correct responses, d-prime reflecting WM performance, and the N2 and P3 components of ERPs. Our results show that there was no effect of tSMS on reaction time. The d-prime was reduced, and the N2 latency was prolonged immediately after tSMS. These findings indicate that tSMS over the left DLPFC affects WM performance and its associated electrophysiological signals, which can be considered an important step toward a greater understanding of tSMS and its use in studies of higher-order cognitive processes.

## 1. Introduction

Non-invasive brain stimulation (NIBS) is an important subject of study in the field of clinical neuroscience, due to its modulatory effects on human brain excitability. In particular, the effectiveness of repetitive transcranial magnetic stimulation (rTMS) and transcranial direct current stimulation (tDCS) in the treatment of clinical conditions has been studied extensively over several decades [1,2]. In addition to these two techniques, transcranial static magnetic field stimulation (tSMS), which uses a neodymium magnet (NdFeB; diameter, 45 mm; height, 30 mm; maximal strength, 765N) [3], has received much attention recently as a less costly and potentially safer alternative. Although several studies report non-significant effects by tSMS [4,5,6], an accumulating body of evidence suggests that tSMS is indeed a powerful NIBS technique that can modulate human functions [7].

Since the first study by Oliviero and colleagues showing reduced motor cortical excitability after the application of tSMS over the motor cortex (M1) [3], the modulatory effects of tSMS on brain function and behavioral performance have been examined in a number of studies. In addition to the M1 excitability [4,5,7,8,9,10,11], for instance, tSMS applied over the sensorimotor area is reported to reduce somatosensory-evoked potentials [12,13,14]. An increase in alpha-band oscillatory power has also been demonstrated with tSMS over the temporal or occipital areas [15,16]. Furthermore, tSMS can modulate the activity of brain regions interconnected with the site of stimulation [17,18,19,20]. In particular, tSMS over the M1 can increase contralateral M1 excitability [19]. In addition, from a behavioral perspective, tSMS over the M1 has been reported to facilitate offline motor learning [21], and to impair pinch force control [22]. Moreover, visual search performance can be modulated with tSMS over the occipital [15] or temporal areas [23], and anticipatory postural control can be impaired with tSMS over the supplementary motor area [24]. Taken together, available evidence indicates that tSMS is capable of modulating human brain function and behavior. However, to our best knowledge, at present, there exists no study investigating the effect of tSMS on working memory (WM) performance.

WM is one of the most common targets for neuromodulation, because it is commonly impaired in individuals with neurological or psychiatric conditions [25,26]. The dorsolateral prefrontal cortex (DLPFC) is a part of the frontal lobes that plays a critical role in executive function [27]. Specifically, the left DLPFC has been considered to be involved in WM processing [28] as disruption of left DLPFC function is associated with impaired WM performance [29,30,31]. On the basis of this evidence, most prior studies examining the effect of NIBS on WM performance targeting the left DLPFC have found that performance can be enhanced by excitatory anodal tDCS [32,33,34] or high-frequency rTMS [33], and impaired by inhibitory cathodal tDCS [30,31] or low-frequency rTMS [30]. Furthermore, a recent meta-analysis by Mancuso and colleagues reported that anodal tDCS over the left DLPFC can facilitate practice-related improvement of WM performance [35]. Given these findings, we hypothesized that tSMS would modulate WM performance or practice-related WM improvement when applied over the left DLPFC.

One task used to measure WM performance is the *n*-back task [36], during which participants are required to respond when a presented visual stimulus is the same as that presented *n* digits previously, and the performance measures can be supplemented by event-related potentials (ERPs). There are two major ERP components observed during the *n*-back task. The first major component that negatively peaks around 200 ms after stimulus onset is called N2 and can be elicited by detection of deviance or mismatch from a perceptual template [37]. In the *n*-back task, this can be reflected by a mismatch between the presented stimulus and a representation held in memory [38]. The second major component that positively peaks after the N2 is called P3 and has been reported to be associated with information processing and decision-making, that is, discrimination of target from non-target [39]. Additionally, P3 can reflect neural activity associated with the process of updating memory [38,40,41].

Accordingly, the purpose of this study was to assess the effect of tSMS over the left DLPFC on WM performance, including practice-related WM improvement. Specifically, we examined the behavioral performance and ERP components during the *n*-back task before, during, and after the tSMS. If tSMS is found to be capable of modulating WM performance, it may potentially be used as a clinical tool to treat frontal brain asymmetry (e.g., unilateral hyperactivity), which is related to psychological and neurological conditions, such as depressive disorders [42].

## 2. Materials and Methods

### 2.1. Participants

Thirteen healthy adults (8 males and 5 females, mean age ± SD = 25.6 ± 3.2 years) participated in this study. Exclusion criteria included psychological and neurological illnesses. None of the participants had metal implants or were under treatment for any conditions. The participants were all right-handed as evaluated by the Edinburg Handedness Inventory [43], and had a normal or corrected-to-normal vision. Written informed consent was obtained after a full explanation of the experiment. This study was approved by the ethics committee of Hiroshima University (No. C-242) and conducted according to the Declaration of Helsinki.

### 2.2. The n-Back Task

**Figure 1:**
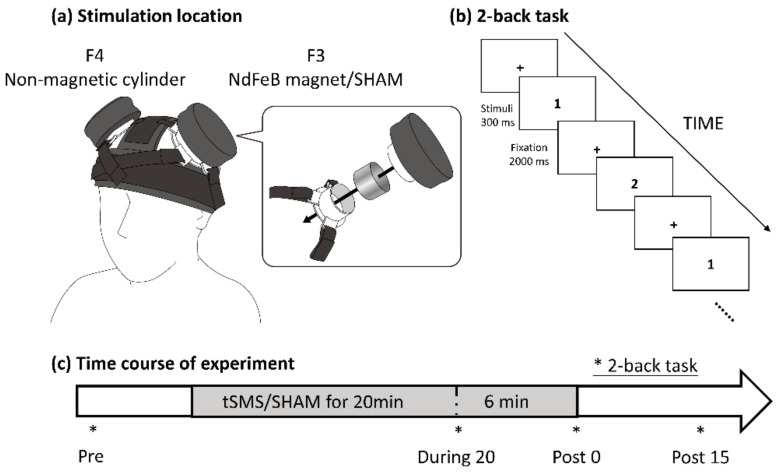
Experimental procedure. The participants received tSMS or SHAM stimulation over the left DLPFC. The magnet or SHAM device was placed on F3, and a non-magnetic cylinder was placed on F4 to counterbalance the weight, using custom-made headgear (**a**). Participants performed the 2-back task (**b**) before (pre), during (during 20), immediately after (post 0), and 15 min after (post 15) tSMS/SHAM stimulation (**c**). The asterisks indicate the time points at which 2-back task was performed.

We used a 2-back version of the *n*-back task in this study (Figure 1). During the task, the participants were presented with a sequence of visual stimuli and required to press a button held in the right hand when the current stimulus matched the one presented two trials previously. The visual stimuli, which consisted of 9 numbers (1–9), were displayed for 300 ms with a 2000 ms interstimulus interval. The task included 150 trials (50 target trials and 100 non-target trials). The visual stimuli were presented using a customized LabVIEW program (National Instruments, Austin, TX, USA).

### 2.3. Study Procedure

A cylindrical NdFeB magnet (diameter, 50 mm; height, 30 mm) with a maximum energy density of 49 MGOe, and a strength of 862 N (88 kg) was used for tSMS (NeoMag Co., Ltd., Ichikawa, Japan), and a non-magnetic stainless-steel cylinder of the same size, weight, and appearance was used as a SHAM device. We used the standard 10–20 system of electrode placement to determine locations of the DLPFC, such that F3 and F4 corresponded to the left and right DLPFCs. The magnet or SHAM device was placed on F3 using custom-made headgear, which was designed by Hiroshima Prefectural Technology Research Institute (Hiroshima, Japan) and manufactured by Fashion Reform Ace (Hiroshima, Japan). Specifically, the magnet or SHAM device was put into a plastic tube case, and the case was covered with a plastic lid and fixed with Velcro straps to the headgear (Figure 1). A non-magnetic stainless-steel cylinder of the same size and weight was placed similarly on F4 to counterbalance the weight. The stimulation was applied for 20 min at rest, followed by an additional 6 min during the 2-back task (26 min total). To avoid carryover effects, each session was conducted on separate days, each at least one day apart. The order of tSMS and SHAM stimulation was counterbalanced across participants.

The participants sat 1 m in front of a monitor, received instructions for the task, and completed one practice session (10 min). Then, they performed the task before, during (20 min after the start of stimulation), immediately after, and 15 min after the tSMS or SHAM stimulation.

### 2.4. Electroencephalography (EEG) Recording

EEG was recorded, while the participants performed the 2-back task, using Ag/AgCl electrodes, from three frontal positions, F3, Fz, and F4, according to the international 10–20 system of electrode placement. The electrodes were referenced to linked earlobes. Electrooculogram (EOG) recordings were made using electrodes placed below the left eye and lateral to the right eye [44]. Electrode impedance was maintained below 10 kΩ. EEG signals were amplified (BA1008; Nihon Santeku, Osaka, Japan) with a band-pass filter of 0.1–100 Hz and sampled at 1 kHz.

### 2.5. Data Analysis

#### 2.5.1. Behavioral Analysis

We assessed reaction times (RT) for correct responses, hit rate (correct response trials/total target trials), false alarm rate (incorrect response trials/total non-target trials), d-prime (a measure of discriminability between the target and non-target), and criterion (a measure of response bias) [45]. The d-prime [46] and criterion were calculated from z transforms of hit rate and false alarm rate, using the following equations: d-prime = z (hit rate) − z (false alarm rate); criterion = −0.5 × [z (hit rate) + z (false alarm rate)].

#### 2.5.2. ERPs Analysis

EEG signals were band-pass filtered between 0.1 and 20 Hz, and divided into epochs of 1000 ms, starting from 100 ms before to 900 ms after the stimulus onset. We excluded trials with errors or misses (i.e., missed target) and epochs with blinks and/or eye movements. The average number of included trials (epochs) was 38 ± 10 for the target condition and 82 ± 17 for the non-target condition. In order to maintain a sufficient signal-to-noise ratio, the minimum number of artifact-free trials per condition being contrasted was set as 12 [47,48]. The minimum number in this study was 12; thus, all conditions from all participants were included in the analysis. The artifact-free epochs were averaged separately for target and non-target conditions. We then detected the peaks of the N2 (200–400 ms) [37], and P3 (150–550 ms) [49] ERP components in the data averaged over the frontal site (Fz, F3, and F4) and estimated their latencies and amplitudes. As N2 and P3 have been estimated to originate from the anterior cingulate cortex (ACC) and its related networks [37,40], they were observed in all the frontal positions; thus, we focused on the average of F3, Fz, and F4 positions, similar to a previous study [50]. The grand average waveform was computed across all participants using the data averaged across trials.

#### 2.5.3. Statistical Analysis

The behavioral and ERP data were confirmed to be normally distributed using a Shapiro-Wilk test (*p* value range, 0.23–0.83). We performed a two-way repeated-measures analysis of variance (ANOVA) to examine the effect of condition (tSMS vs. SHAM) and time (pre, during 20, post 0, and post 15) on behavioral data (RT, d-prime, and criterion). We also performed a three-way repeated-measures ANOVA to examine the effect of condition, time, and trial type (Target vs. Non-target) on ERP data (N2 and P3 amplitudes and latencies). Post hoc paired t-tests were performed using a Bonferroni correction. The statistical analyses were conducted using SPSS Statistics software version 21 (IBM, Armonk, NY, USA). The significance level was set at *p* < 0.05.

## 3. Results

### 3.1. Behavioral Data

**Figure 2:**
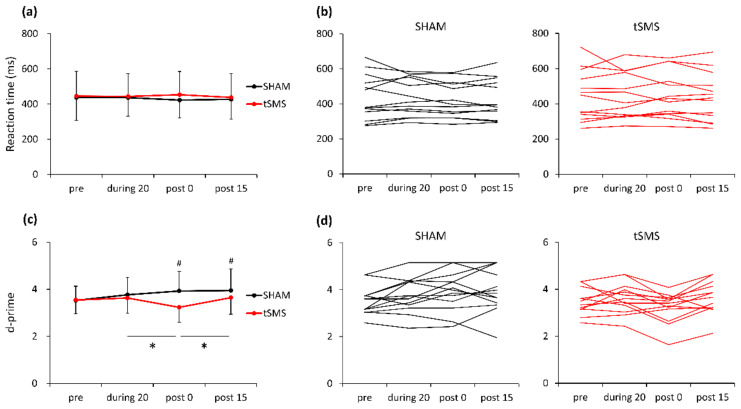
Mean and individual values of reaction time (**a**,**b**) and d-prime (**c**,**d**). * *p* < 0.05 during 20 vs. post 0 and post 0 vs. post 15, # *p* < 0.05 tSMS vs. SHAM at post 0 and post 15.

Mean RT, hit rate, false alarm rate, d-prime, and criterion are presented in Table 1. A two-way repeated-measures ANOVA revealed no main effect of condition or time or their interaction on RT (Figure 2a,b). In contrast, there were significant main effects of condition (F [1,12] = 6.411, *p* = 0.026, partial η^2^ = 0.348) and time (F [3,36] = 4.440, *p* = 0.031, partial η^2^ = 0.571) and their interaction (F [3,36] = 6.103, *p* = 0.013, partial η^2^ = 0.647) on d-prime. Post hoc analyses revealed that d-prime significantly decreased with tSMS over F3 from during 20 to post 0 (mean difference = −0.397, *p* = 0.038), and significantly increased from post 0 to post 15 (mean difference = 0.413, *p* = 0.035). Also, there was a significant difference in d-prime between tSMS and SHAM conditions at post 0 (mean difference = −0.695, *p* = 0.003) and at post 15 (mean difference = −0.304, *p* = 0.043) (Figure 2c,d). There was no significant main effect of condition or time or their interaction on criterion (*p* > 0.05).

### 3.2. ERP Data

**Figure 3:**
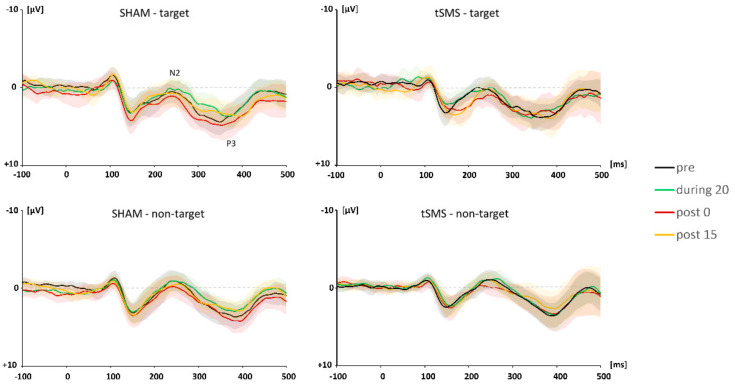
Grand average ERP waveforms for target and non-target trials. The shaded area indicates 95% confidence intervals.

Figure 3 depicts the grand average waveforms for the target and non-target trials shown separately for each time period (pre, during 20, post 0, and post 15).

#### 3.2.1. N2 Component

A three-way repeated-measures ANOVA revealed main effects of trial type (F [1,12] = 12.943, *p* = 0.004, partial η^2^ = 0.906) and time (F [1,12] = 5.920, *p* = 0.014, partial η^2^ = 0.860) on N2 amplitude. Specifically, N2 amplitude for non-target trials was larger than that for target trials (*p* < 0.001). A post hoc analysis indicated that N2 amplitude at post 0 was significantly smaller than that at pre (*p* = 0.011) and during 20 (*p* < 0.001).

**Figure 4:**
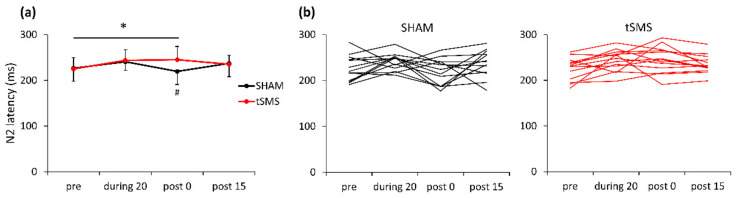
Mean (**a**) and individual (**b**) N2 latencies during the 2-back task. * *p* < 0.05 pre vs. post 0, # *p* < 0.05 tSMS vs. SHAM at post 0.

A three-way repeated-measures ANOVA revealed significant interactions of condition × time (F [3,36] = 7.018, *p* = 0.008, partial η^2^ = 0.912) and of trial type × condition × time (F [3,36] = 4.609, *p* = 0.028, partial η^2^ = 0.707) for N2 latency. For the target trials, there was a significant simple main effect of time (F [3,36] = 4.114, *p* = 0.039, partial η^2^ = 0.565) and a significant simple interaction of condition × time (F [3,36] = 7.018, *p* = 0.008, partial η^2^ = 0.690). A post hoc analysis revealed that N2 latency was significantly prolonged with tSMS over F3 from pre to post 0 (mean difference = 19.231, *p* = 0.001). At post 0, there was a significant difference in N2 latency between tSMS and SHAM (mean difference = 24.538, *p* = 0.031) (Figure 4).

#### 3.2.2. P3 Component

A three-way repeated-measures ANOVA revealed no main effect of trial type or time or their interaction on P3 amplitude. There was a main effect of trial type (F [1,12] = 20.531, *p* = 0.001, partial η^2^ = 0.986) on P3 latency, indicating that P3 latency was faster in target than non-target trials.

## 4. Discussion

In this study, we investigated the effect of tSMS over the left DLPFC on WM performance and associated ERPs using the 2-back task. We found WM performance to be impaired and N2 latency to be prolonged with tSMS immediately after its removal. These findings suggest that tSMS over the left DLPFC is capable of modulating WM performance and its associated electrophysiological signals.

### 4.1. The Effect of tSMS on Behavioral Performance

Although the precise mechanism of how tSMS modulates neural activity is not currently fully understood, a recent review confirmed that the static magnetic fields (SMFs) created by the NdFeB magnet used for tSMS have a sufficient capacity to influence cellular systems [51]. Specifically, radial pair recombination and biomolecule reorientation by diamagnetic anisotropy effects result in susceptibility of biomolecules, intracellular structural modifications, and changes in enzymatic reactions [51]. Rosen also suggested that SMFs can induce reorientation of membrane phospholipids by diamagnetic anisotropy [52], which then deforms ion channels and alters their functions [53]. At present, however, no definite conclusions have been made on this subject. On the other hand, in humans it has been demonstrated in multiple studies that tSMS can reduce cortical excitability in various brain areas [3,7,8,9,10,11,12,13,14]. Therefore, it is likely that tSMS reduced the excitability of the left DLPFC, which modulated the WM performance in the present study.

While WM performance was impaired by the tSMS over the left DLPFC immediately after its removal (post 0) and 15 min after the stimulation, the tSMS effect was not observed for RT, indicating that there was no speed-accuracy tradeoff, in which faster responses were made with less accuracy. This finding is consistent with previous studies investigating the effect of NIBS over the left DLPFC on WM. Specifically, anodal tDCS over the left DLPFC was found to enhance WM performance, but did not affect RT [54]. Also, low-frequency rTMS (1 Hz) over the left DLPFC has been shown to affect WM performance, but not RT [26,55]. Since the DLPFC plays an important role in WM maintenance and is continuously activated during the delay period of WM tasks (for the maintenance and update of task-relevant information) [27], tSMS may have impaired this function of the DLPFC. The reason we observed a reduction in WM performance only after tSMS removal (post 0) and not during the tSMS is hard to interpret; however, we propose two possibilities: First, since the stimulation duration was longer at the post-0 session (approximately 26 min) than at the during-20 session (20 min), a longer stimulation duration may have been necessary to induce the effect on WM performance. Second, as mentioned in a review by Hill and colleagues [32], homeostatic control of cortical excitability and inhibition may be involved in the online and offline effects of NIBS. The authors interpreted their findings of an online tDCS effect on WM in neuropsychiatric patients and offline tDCS effect on WM in healthy individuals as indicating that abnormal balance between cortical excitation and inhibition in patients results in greater vulnerability to stimulation at the initial stage and that the impact of stimulation is reduced in healthy individuals with optimal homeostatic control of cortical excitation and inhibition. Although speculative, the offline tSMS effect found in this study may have been driven by a similar mechanism. Further studies are clearly warranted to better understand the mechanism of tSMS online and offline effects.

### 4.2. The Effect of tSMS on ERPs

#### 4.2.1. The N2 Component

The N2 component, the second negative peak in an ERP waveform, is considered to originate from the ACC and can be divided into two subcomponents: Frontocentral and posterior [37]. Our ERP data correspond to the frontocentral (anterior) N2, which is sensitive to mismatch detection and cognitive control. During WM tasks, the ACC can act as a controller, evaluating the need for behavioral adjustments based on the received information [56,57], while the DLPFC plays an important role in WM maintenance and control of other WM-related brain regions [55,58]. Consistent with these views, the anterior N2 amplitude is commonly larger in non-target (mismatch) than target trials (match) in the *n*-back task [37], and we identified such a difference in this study. Meanwhile, when comparing individuals with high WM performance and those with low WM performance, the N2 amplitude itself does not show a difference; instead, N2 latency has been shown to be prolonged in individuals with low WM performance [38,59]. In the present study, we similarly observed impaired WM performance and prolonged N2 latency immediately after tSMS over the left DLPFC. Given the role N2 plays in the process of WM, our findings may imply that the tSMS influenced the WM maintenance by the left DLPFC, which interfered with the quality of information and caused difficulties evaluating the information received from the left DLPFC.

#### 4.2.2. The P3 Component

The anteriorly distributed P3, which positively peaks following the N2 in an ERP waveform, is also considered to be generated by the ACC and its connections [40]. P3 can be a measure of decision-making and memory updating [60], and its amplitude depends on the number of processing resources given to these operations [61]. In the *n*-back task, the P3 was found to be larger and more prolonged in individuals with low WM performance than in those with high WM performance [38], which can be interpreted as reflecting that individuals with low WM performance allocated more cognitive resources to determine whether the present stimulus matched a representation held in memory. Indeed, it has been hypothesized that a failure to produce an electrophysiological signal detecting the match/mismatch (N2) induces a greater load on the subsequent decision-making process (P3) [38]. In the present study, however, we did not observe any effect of tSMS on the P3 amplitude or latency, despite the finding of a prolonged N2 latency. Although the exact mechanism cannot be explained, we hypothesize that the tSMS over the left DLPFC reduced the quality of information held in WM, which had the participants miss targets instead of allocating cognitive resources to decision-making, given that the number of misses was much larger than that of errors (average miss: 10 times, average error: 1.15 times). Nevertheless, our findings indicate that tSMS is capable of modulating WM performance and its associated electrophysiological signal (N2), suggesting that tSMS may be useful for studying the neural bases of higher-order cognitive processes.

### 4.3. Potential Clinical Application

Major depression disorder (MDD), which is characterized by persistent low mood, loss of interest and/or enjoyment, and reduced energy, is associated with a loss of quality of life and high social and economic costs. Although its pathophysiology is not yet fully understood, a variety of treatments, such as antidepressant medication and psychological therapies, are included in MDD treatment guidelines [62,63]. However, they are effective only in some patients, and response rates in MDD treatment have been reported to be about 50% [64]. Thus, the development of new effective treatment options is highly desirable. To pursue novel MDD treatments, many studies in the last three decades have examined the efficacy of NIBS. As MDD is associated with neural and metabolic activity asymmetry in the two prefrontal areas, with the right side hyperactivated and the left side hypoactivated, NIBS can correct this activity asymmetry [65,66,67]. Specifically, excitatory anodal tDCS or high-frequency rTMS can be applied to the left DLPFC to enhance its activity, or inhibitory cathodal tDCS or low-frequency rTMS can be applied to the right DLPFC to reduce its activity. Recent meta-analyses have shown that excitatory and inhibitory NIBS techniques are both effective in MDD treatment [68,69]. Given the findings of the present study, tSMS seems to have the potential to be used in the treatment of individuals with MDD by reducing the hyperactivity of the right DLPFC. Moreover, the magnet used for tSMS is inexpensive (approximately 200 US dollars), and its operation does not require special training; thus, tSMS may be used as a tool for home rehabilitation. Whether tSMS is effective in treating MDD should be investigated in future studies.

## 5. Conclusions

The present study investigated the effect of tSMS over the left DLPFC on WM performance and associated ERPs. We found that tSMS impaired the WM performance and prolonged the N2 latency. These findings suggest that tSMS can affect the neural activity of the DLPFC, and thus, may be useful for studying the neural bases of higher-order cognitive processes.

## Figures and Tables

**Table 1:** Reaction time (RT), hit rate, false alarm rate, d-prime, and criterion (mean ± SD).

		SHAM	tSMS
RT (ms)	pre	436.87 ± 128.74	445.16 ± 141.71
	during 20	436.73 ± 106.05	443.59 ± 129.99
	post 0	422.82 ± 101.02	453.31 ± 132.31
	post 15	426 ± 111.88	437.93 ± 135.18
Hit rate	pre	0.86 ± 0.10	0.83 ± 0.14
	during 20	0.87 ± 0.13	0.86 ± 0.12
	post 0	0.87 ± 0.15	0.80 ± 0.15
	post 15	0.87 ± 0.15	0.85 ± 0.15
False alarm rate	pre	0.01 ± 0.01	0.01 ± 0.01
	during 20	0.01 ± 0.01	0.01 ± 0.00
	post 0	0.01 ± 0.00	0.01 ± 0.01
	post 15	0.01 ± 0.01	0.01 ± 0.01
d-prime	pre	3.53 ± 0.60	3.54 ± 0.59
	during 20	3.77 ± 0.74	3.63 ± 0.64 *
	post 0	3.93 ± 0.84	3.23 ± 0.64 #
	post 15	3.95 ± 0.92	3.64 ± 0.70 *
criterion	pre	0.57 ± 0.28	0.67 ± 0.29
	during 20	0.50 ± 0.37	0.59 ± 0.25
	post 0	0.54 ± 0.36	0.70 ± 0.27
	post 15	0.51 ± 0.39	0.62 ± 0.28

* *p* < 0.05 during 20 vs. post 0 and post 0 vs. post 15, # *p* < 0.05 tSMS vs. SHAM at post 0 and post 15.

## Data Availability

Data available on request.
